# Role of *Cr*RLK1L Cell Wall Sensors HERCULES1 and 2, THESEUS1, and FERONIA in Growth Adaptation Triggered by Heavy Metals and Trace Elements

**DOI:** 10.3389/fpls.2017.01554

**Published:** 2017-09-07

**Authors:** Julia Richter, Marie Ploderer, Gaëlle Mongelard, Laurent Gutierrez, Marie-Theres Hauser

**Affiliations:** ^1^Department of Applied Genetics and Cell Biology, University of Natural Resources and Life Sciences, Vienna Vienna, Austria; ^2^Centre de Ressources Régionales en Biologie Moléculaire, Université de Picardie Jules Verne Amiens, France

**Keywords:** cadmium, copper, lead, nickel, zinc, alkalinization, root growth, hypocotyl elongation

## Abstract

Cell walls are not only a protective barrier surrounding protoplasts but serve as signaling platform between the extracellular environment and the intracellular physiology. Ions of heavy metals and trace elements, summarized to metal ions, bind to cell wall components, trigger their modification and provoke growth responses. To examine if metal ions trigger cell wall sensing receptor like kinases (RLKs) of the *Catharanthus roseus* RLK1-like (*Cr*RLK1L) family we employed a molecular genetic approach. Quantitative transcription analyses show that *HERCULES1 (HERK1)*, *THESEUS1 (THE1)*, and *FERONIA (FER)* were differently regulated by cadmium (Cd), nickel (Ni), and lead (Pb). Growth responses were quantified for roots and etiolated hypocotyls of related mutants and overexpressors on Cd, copper (Cu), Ni, Pb, and zinc (Zn) and revealed a complex pattern of gene specific, overlapping and antagonistic responses. Root growth was often inversely affected to hypocotyl elongation. For example, both *HERK* genes seem to negatively regulate hypocotyl elongation upon Cd, Ni, Zn, and Pb while they support root growth on Cd, Cu, and Ni. The different *THE1* alleles exhibited a similar effect between roots and hypocotyls on Ni, where the loss-of-function mutant was more tolerant while the gain of function mutants were hypersensitive indicating that *THE1* is mediating Ni specific inhibition of hypocotyl elongation in the dark. In contrast hypocotyl elongation of the knock-out mutant, *fer-4*, was hypersensitive to Ni but exhibited a higher tolerance to Cd, Cu, Pb, and Zn. These data indicate an antagonistic action between THE1 and FER in relation to hypocotyl elongation upon excess of Ni. FERs function as receptor for rapid alkalinization factors (RALFs) was tested with the indicator bromocresol purple. While *fer-4* roots strongly acidified control and metal ion containing media, the etiolated hypocotyls alkalized the media which is consistent with the already shorter hypocotyl of *fer-4*. No other *CrRLK1L* mutant exhibited this phenotype except of the THE1:GFP overexpressor on Ni suggesting that THE1 might be involved in Ni induced and hypocotyl specific RALF signaling and growth regulating pathway. Overall, our findings establish a molecular link between metal ion stress, growth and the cell wall integrity sensors of the *Cr*RLK1L family.

## Introduction

Heavy metals and trace elements in excess as well as deficiency in soils impose a major challenge to plant growth in general and crop productivity. From studies of metal ion tolerant and hyperaccumulating metallophytes as well as sensitive plants such as *Arabidopsis thaliana* we learnt that excess of metal ions induce a complex network of responses thoroughly reviewed in DalCorso et al. (2013) and Singh et al. (2016). The regulation of metal ion homeostasis involves increased biosynthesis of chelators as well as efflux and influx transporters essential for compartmentalization. Another strategy to prevent or reduce uptake of metals is by restricting metal ions to the cell wall. The cell wall, rich in functional carbohydrate originated carboxyl and hydroxyl as well as protein derived sulfhydryl and histidyl groups plays a key role in the immobilization of metal ions (Krzesłowska, 2011). Metal ion immobilization in the cell wall is mainly mediated by the pectic polysaccharide homogalacturonan (Pelloux et al., 2007). The capacity of binding is enhanced by the activity of cell wall-associated pectin-methylesterases (PMEs) or pectin-acetylesterase (PAEs) exposing free negatively charged carboxyl groups. They form salt bridges contributing to the mechanical strength of cell walls. Mainly calcium (Ca) is used in theses so called “egg boxes” but other metals ions with often higher affinities such as aluminum (Al), copper (Cu), lead (Pb), zinc (Zn), cadmium (Cd), cobalt (Co), nickel (Ni), barium (Ba), strontium (Sr), manganese (Mn), magnesium (Mg), iron (Fe), chromium (Cr), and mercury (Hg) have been shown to bind to de-esterified pectins (Dronnet et al., 1996; Kartel et al., 1999; Meychik et al., 2011). Expression studies in diverse plants show that cell wall modifying enzymes were upregulated upon metal ion stress (Hassinen et al., 2007; Konlechner et al., 2013). Also PME activities and structural modifications of pectins change upon metal ion treatments and are related to metal ion tolerance and growth responses (Paynel et al., 2009; Douchiche et al., 2010; Weber et al., 2013; Yang et al., 2013; El-Moneim et al., 2014; Muschitz et al., 2015; Geng et al., 2017) and reviewed in Parrotta et al. (2015).

Pectin, highly de-esterified pectates and their degradation products are important components of the cell wall integrity pathways (Wolf et al., 2012; Voxeur and Höfte, 2016). It has been shown that pectins and oligogalacturonic acids bind to the extracellular domain of and activate WAK1 and WAK2, members of the *WALL ASSOCIATED KINASES* gene family (Decreux and Messiaen, 2005; Decreux et al., 2006; Kohorn et al., 2006). Furthermore, WAKs and WAK-LIKE receptors (WAKLs) are involved in the regulation of cell expansion and responses to metal ions (Lally et al., 2001; Wagner and Kohorn, 2001; Hou et al., 2005). Apart from WAK(L)s another pectin-associated kinase, proline-rich extensin-like receptor kinase 4 (PERK4), is involved in drought stress mediated growth responses (Bai et al., 2009). Also, several leucine-rich repeat (LRR) receptor like kinases (RLKs) are involved in cell wall integrity pathways related to pathogen signaling and are necessary for the synthesis of cell wall components upon high sucrose and NaCl conditions (Xu et al., 2008; Engelsdorf and Hamann, 2014).

In this study we focused on the *Catharanthus roseus* RLK1-like (*Cr*RLK1L) protein family. The *Cr*RLK1L family consists of 17 members which all share an extracellular domain homologous to the animal malectin protein with putative carbohydrate binding capacity. Therefore the *Cr*RLK1L malectin-like domains might bind to oligo- or polysaccharides from cell wall polymers, by-products of cell wall degradation, or membrane-associated or secreted glycosylated proteins (Schallus et al., 2008; Boisson-Dernier et al., 2011). Recently it has been demonstrated that the *Cr*RLK1L member, FERONIA (FER), is the receptor for peptides of the RAPID ALKALINIZATION FACTOR family (Haruta et al., 2014) while the ligands for the other members are still disclosed. *Cr*RLK1L proteins play diverse roles during fertilization (Escobar-Restrepo et al., 2007; Boisson-Dernier et al., 2009; Miyazaki et al., 2009) but are also important during vegetative development and in plant pathogen interactions (Bai et al., 2014; Gachomo et al., 2014; Nissen et al., 2016; Stegmann et al., 2017).

Here, we focus on the four *Cr*RLK1L members which regulate cell expansion and growth of seedlings: FER, THESEUS1 (THE1), HERCULES1 (HERK1), and HERK2 (Hématy et al., 2007; Guo et al., 2009a,b; Merz et al., 2017) and their role in growth responses on elevated concentrations of Cd, Cu, Ni, Pb, and Zn. With the help of loss- and gain-of-function alleles, a complex pattern of gene specific, overlapping and antagonistic reactions was revealed. Root growth was often inversely affected to hypocotyl elongation. Antagonistic roles of the two *HERK* genes and *FER* versus THE1 were discovered in relation to root growth on Cu, in relation to hypocotyl elongation on Pb and Zn, and between *THE1* and *FER* on Ni. The effect of metal ions on the acidification ability was evaluated with bromocresol purple indicator medium. While *fer-4* roots strongly acidified control and metal ion containing media, etiolated hypocotyls alkalized the media which is consistent with the *fer-4* elongation defect. No other *CrRLK1L* mutant exhibited this phenotype except for the THE1:GFP overexpressor on Ni suggesting that THE1 might be involved in an Ni induced and hypocotyl specific rapid alkalinization factor (RALF) signaling and growth regulating pathway. Overall, our findings establish a molecular link between metal ion stress, growth and the cell wall integrity sensors of the *Cr*RLK1L family.

## Materials and Methods

### Plant Material

Col-0 was used as wild type. T-DNA mutants were all in Col-0 background and provided either by the SALK collection (Alonso et al., 2003), *herk1* (SALK_008043), *herk2.1* (SALK_105055), *herk2.2* (SALK_107146), the SAIL collection (Sessions et al., 2002), *the1-4* (SAIL_683_H03), and the GABI-Kat collection (Kleinboelting et al., 2012), *fer-4* (GABI_GK106A06). The loss-of-function allele, *the1-6*, was isolated in a suppressor screen of *ctl1-1/pom* and described in Merz et al. (2017). The THE1:GFP overexpression line was described in Hématy et al. (2007).

### Growth Conditions

Seeds were surface-sterilized in 5% sodium hypochlorite and rinsed three times with sterile deionized water and then transferred to nutrient agar medium plates containing 1/10 strength Hoagland salts, 1% (w/v) sucrose and 1% (w/v) agar (Duchefa). For metal ion treatments metal salts were added after autoclaving to final concentrations of 10 μM CdCl_2_, 5 μM CuSO_4_, 15 μM NiSO_4_, 100 μM Pb(NO_3_)_2_, and 100 μM ZnSO_4_. After 2 days of imbibition at 4°C in the dark, plates were vertically incubated in a growth chamber at 22°C with constant light (80 μmol m^2^ s^-1^). For measurements of etiolated hypocotyls, plates were wrapped in aluminum foil after exposure to light for 5 h. Isoxaben treatment for gene expression analyses was essentially done as described in Merz et al. (2017). Ni treatment for gene expression analyses was done for 6 h on 4 days after germination (dag) etiolated seedlings.

### Bromocresol Purple (BCP) Plates

For pH assays sterilized seeds were placed on nutrient agar plates as described above supplemented with 150 μM BCP and metal ions in indicated concentrations. Plates were scanned on day 5 after germination.

### Growth Analysis

The plates were scanned on days 3, 4, and 5 after germination for root growth and on day 5 after germination for hypocotyl measurements. The lengths were evaluated with the ImageJ software by freehand tracking.

### RNA Isolation and cDNA Synthesis

Total RNA of 10–12 day old seedlings germinated and grown on control and metal ion supplemented medium were snap frozen and isolated using a LiCl/CTAB method. After grinding roughly 100 mg frozen seedlings 1 mL of pre-heated RNA extraction buffer (2% [w/v] hexadecyltrimethylammonium bromide, CTAB; 2% [w/v] polyvinylpyrrolidone, PVP; 100 mM Tris/HCl pH 8.0; 25 mM EDTA; 2 M NaCl; 0.5 g/L spermidine and 2.7% [v/v] 2-mercaptoethanol) was added, mixed and incubated at 65°C for 5 min. CTAB was removed through two times separation with 1 mL of ice-cold chloroform: isoamylalcohol (24:1) and centrifugation at 4°C. RNA in the supernatant was precipitated with 250 μL 10 M LiCl at 4°C for more than 1.5 h. After centrifugation and EtOH washes the pellet was dissolved in 20 μL RNase free water and stored at -80°C. RNA was quantified with the Qubit (Invitrogen) and the NanoDrop systems. RNA integrity was controlled on Agilent 2100 Bioanalyzer using RNA Nanochip. RNA samples were treated with DNaseI using the TURBO DNA-*free*^TM^ kit (Ambion). cDNAs were synthesized from 4 μg total RNA using the Transcriptor reverse transcriptase (Roche) with 500 pmol of oligo(dT)_18_ primer. The reaction was stopped at 85°C for 5 min without further treatment according to the manufacturer’s instructions. cDNA was diluted 20 times with distilled water and tested by PCR using specific primers flanking an intron sequence to confirm the absence of genomic DNA contamination. cDNA synthesis for the *THE1* downstream gene expression was done as described in Merz et al. (2017).

### Reverse Transcription (RT)-qPCR Data Analysis

*AP2M* (*ADAPTOR PROTEIN-2 MU-ADAPTIN*) has been validated to be the most stably expressed gene among eight tested and was used to normalize the RT-qPCR data (Gutierrez et al., 2012). CT and PCR efficiency (E) values were used to calculate expression using the formula E_T_(^CT^_ctr_^-CT^_m_)/E_R_(^CT^_ctr_^-CT^_m_), where T is the target gene and R is the reference gene, CT is the crossing threshold value, m refers to cDNA from the metal ion treated seedlings, and ctr refers to cDNA from the control medium. All RT-qPCR results presented are means from three independent biological replicates and for each independent biological replicate, the relative transcript amount was calculated as the mean of three technical replicates, using the method for calculation of SE values in relative quantification recommended by Rieu and Powers (2009). RT-qPCR for the *THE1* downstream gene expression was done as described in Merz et al. (2017). Primers used for the RT-qPCRs are summarized in Supplementary Table 2.

## Results And Discussion

### Members of the *Cr*RLK1L Family Are Induced upon Metal Ion Exposure

**FIGURE 1:**
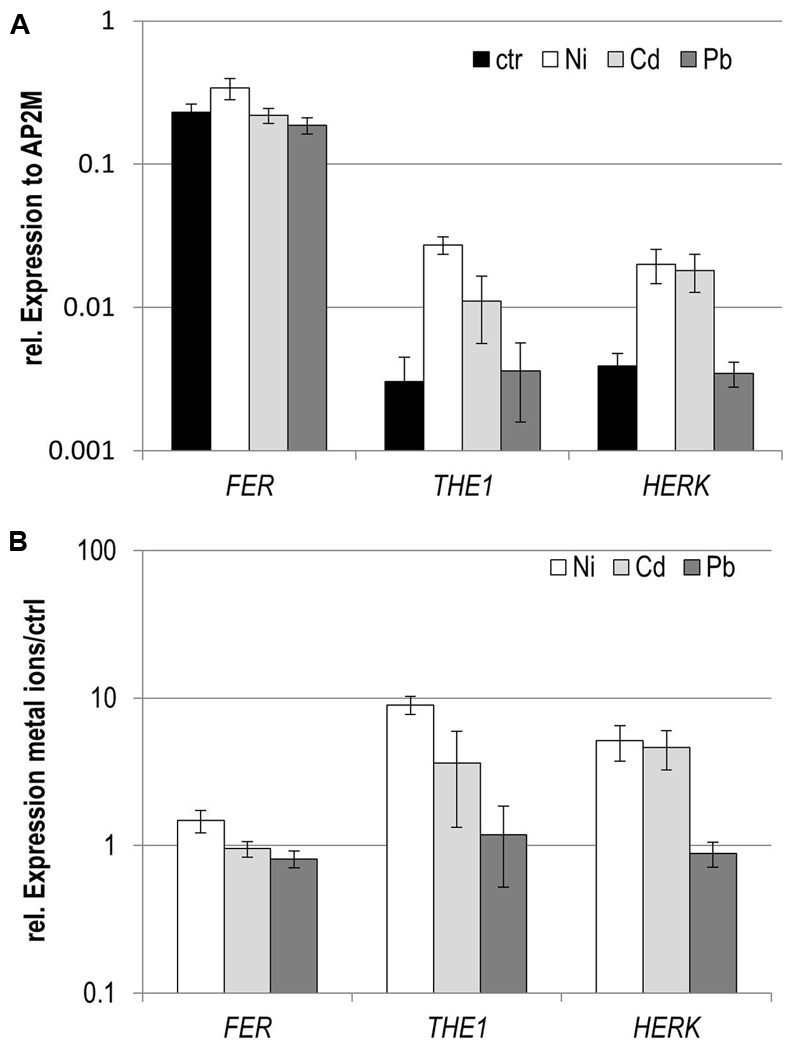
Expression analyses of *Cr*RLK1L members in seedlings exposed to different metal ions to different metal ions. **(A)** Expression normalized to the reference gene *AP2M*, **(B)** expression relative to the control conditions. Data are mean and SE of three biological experiments with each three technical repeats.

In an expression survey for genes responsive to the metal ions Cd, Ni, and Pb in seedlings, the *Cr*RLK1L members *THE1*, *FER*, and *HERK1* exhibited a remarkable expression pattern. All three were induced by Ni, only slightly for *FER* but strongly for HERK1 and *THE1* (**Figure 1**). Pb did not induce any transcriptional regulation whereas Cd triggered upregulation of *THE1* and *HERK1*.

### Root and Etiolated Hypocotyl Growth Differ in Response to Metal Ion Medium

Growth and cell expansion responses of germinating Arabidopsis seedlings to elevated metal ion concentrations depend strongly on the composition of the growth medium and on the cation exchange capacity of the agar. The high cation concentration such as provided by the frequently used 1x Murashige and Skoog medium (Murashige and Skoog, 1962) interferes with the uptake of additional metal ions. On 1x MS medium 400 μM Zn and 40 μM Cd were needed to observe a slight root length reduction for Zn and a 30% reduction for Cd (Kobae et al., 2004). A comparable experiment with germinating seedlings on 1/10 Hoagland medium supplemented with metal ions needed only 50 μM Zn for a 43.2% and 2 μM Cd for a 57.6% root growth inhibition (Tennstedt et al., 2009). Since the MS medium is also Fe- satiated and Cu-deficient all metal ion experiments were performed on 1/10 Hoagland medium (Supplementary Table 1).

Root growth depends on two parameters, the rate of cell division and cell expansion, while dark stimulated hypocotyl growth depends solely on cell expansion. To separate the cell division from cell expansion effects, root growth was determined on seedlings germinating and grown for 5 days in the light and hypocotyl elongation was assessed on 5 dag dark-grown seedlings. In both sets of experiments identical 1/10 Hoagland media supplemented with either 10 μM Cd, 5 μM Cu, 15 μM Ni, 100 μM Pb, or 100 μM Zn were used.

**FIGURE 2:**
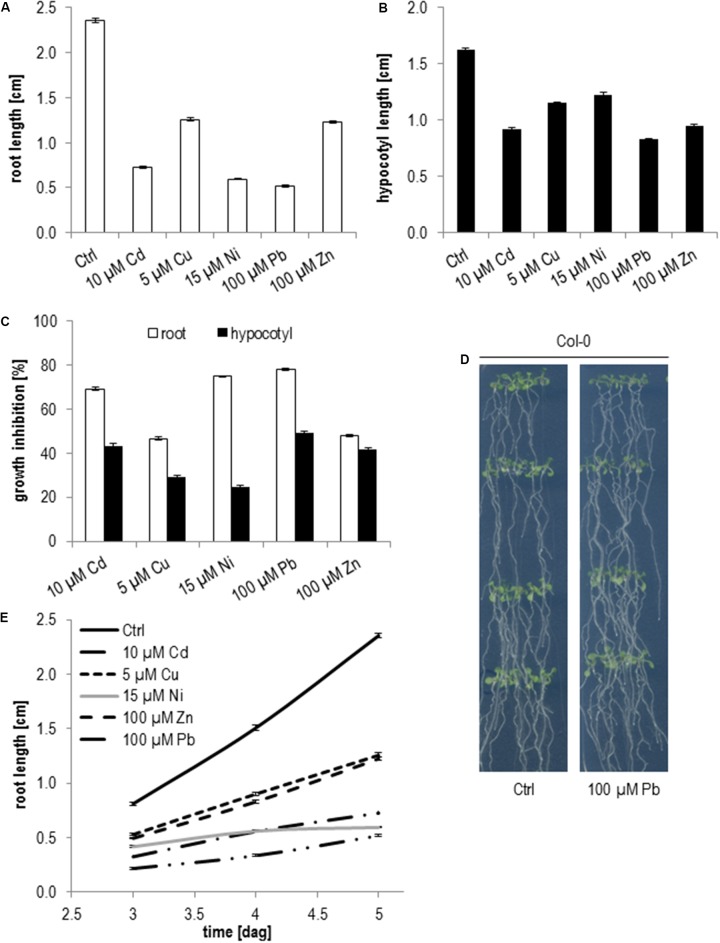
Root growth and etiolated hypocotyl elongation of Col-0 seedlings upon elevated metal ion concentrations. **(A)** Root length 5 days after germination, **(B)** hypocotyl length after 5 days of germination in the dark, **(C)** growth inhibition relative to control medium, **(D)** root growth between days 3 and 5 after germination, **(E)** seedlings on control and Pb 11 days after germination. Shown are mean and SE of at least three biological replicates with 20 seedlings each.

Generally, hypocotyl elongation is less sensitive to metal ions (**Figures 2A–C**). While on 10 μM Cd root growth was reduced by 70% in relation to control medium the growth inhibition of hypocotyls was only 43%. Similar results were obtained for 5 μM Cu with 46% root and 30% hypocotyl inhibition, 100 μM Zn with 47% root and 41% hypocotyl inhibition, 15 μM Ni with 74% root and 25% hypocotyl inhibition and 100 μM Pb with 78% root and 49% hypocotyl inhibition (**Figure 2C**). The most dramatic difference between root and hypocotyl growth inhibition was seen for Ni followed by Pb and Cd (**Figure 2C**) indicating that these metal ions might influence cell division more severely than cell expansion. It might also be possible that cell expansion in the root is differently affected by these metal ions than in etiolated hypocotyls. The difference between root and hypocotyl growth is particularly true for Ni where root growth rate was continuously declining and nearly stopped 5 days after germination (dag) (**Figure 2D**). Pb shows the opposite effect and root growth recovered starting at 5 dag and roots reached nearly the same length as wild type at 11 dag (**Figure 2E**).

Apart of the dissimilar effects of metal ions on cell division and cell expansion another explanation for the differing sensitivities between root and hypocotyl growth would be that metal ion transport to hypocotyls is less effective. Data on metal ion concentrations in separated roots and hypocotyls and quantifications of the cell division rate and cell sizes would resolve these possibilities. Although the results of these analyses would contribute to the understanding of the root/hypocotyl response differences, the focus of this study was to determine if members of the *Cr*RLK1L family are involved in mediating growth responses to metal ions.

### HERCULES1 and 2 Negatively Regulate Hypocotyl Elongation on Cd, Ni, Zn, and Pb While They Support Root Growth on Cd, Cu, and Ni

**FIGURE 3:**
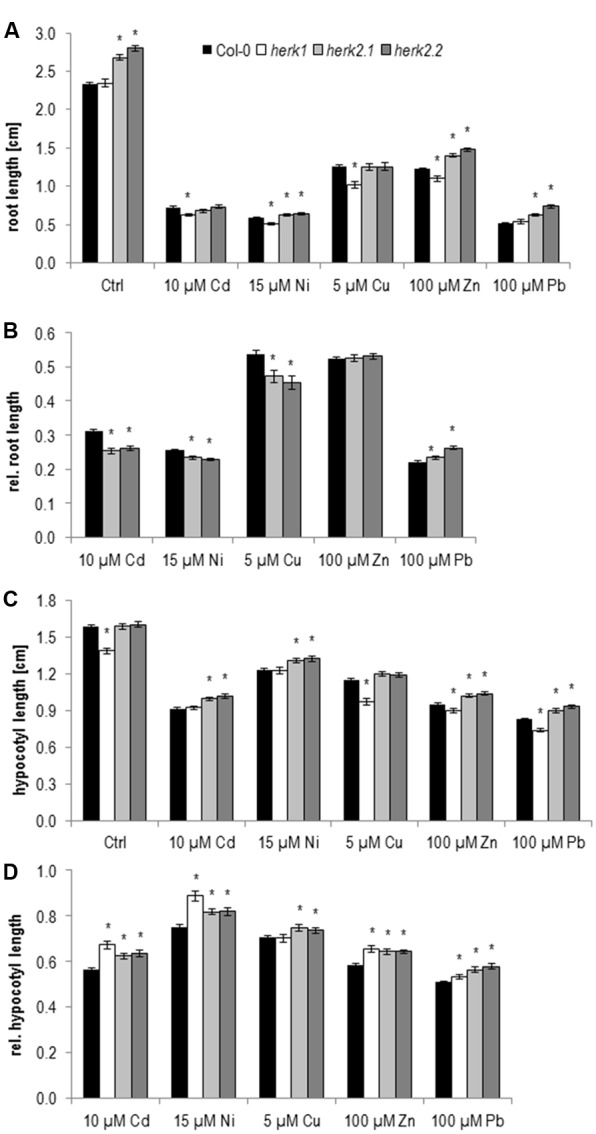
Root growth and etiolated hypocotyl elongation of *herk1* and *herk2* alleles upon elevated metal ion concentrations at 5 days after germination. **(A)** Absolute root lengths, **(B)** relative root lengths of *herk2* alleles, **(C)** absolute hypocotyl lengths, **(D)** relative hypocotyl lengths. Shown are mean and SE of at least three biological replicates with 20 seedlings each. Stars indicate significant differences according to Student’s *t*-tests with ^∗^*p* < 0.05 to wild type **(A,C)**, wild type and control medium **(B,D)**.

Previous growth assays with single mutants impaired in *HERK1* and *HERK2* expression did not show any obvious growth phenotype. But the mutants developed strong cell elongation defects in combination with the *the1-4* allele (Guo et al., 2009a,b). However *the1-4* turned out to be a hypermorphic allele. Thus the growth defects of the double and triple mutants indicate that *HERK1* and *HERK2* act antagonistically and not redundantly to *THE1* (Merz et al., 2017). On 1/10 Hoagland control medium, roots of *herk1* seedlings grow similarly to wild type in contrast to both *herk2* alleles, which exhibited a significantly faster root growth (**Figure 3A** and **Table 1**).

**Table 1:** Summary of the significant growth and elongation responses in comparison to wild type.

Genotype	ctrl		Cd		Cu		Ni		Pb		Zn	
Organ	r	h	r	h	r	h	r	h	r	h	r	h
*herk1*	=	↓	↓	↑	↓	=	↓	↑	=	↑	↓	↑
*herk2.1*	↑	=	↓	↑	↓	=	↓	↑	↑	↑	=	↑
*herk2.2*	↑	=	↓	↑	↓	=	↓	↑	↑	↑	=	↑
*the1-6*	↓	=	↓	=	=	=	↑	↑	=	↓	↓	↓
*the1-4*	=	↓	↑	=	↓	=	↑	↓	=	↑	=	↑
THE1:GFP	↑	↓	↑	↑	↓	↑	↓	↓	=	=	=	↑
*fer-4*	↓	↓	=	↑	↓	↑	=	↓	↑	↑	↓	↑

*Data of roots and hypocotyls are indicated with r and h, respectively. In case growth on control medium was already significantly different between wild type and the CrRLK1L mutants the significances of the normalized data are employed*.

On metal ion supplemented medium root growth of *herk1* seedlings is stronger inhibited upon Cd, Ni, Cu, and Zn than wild type. Since the two *herk2* alleles already grow faster on control medium, analyzing the effect of metal ions on these mutants by only looking at the absolute root length data might be misleading. Therefore, their root growth on metal ions was normalized to control conditions (**Figure 3B** and **Table 1**). These analyses revealed that the apparent higher tolerance to Ni, and Zn diminished and only the reduced lead response remained while an enhanced Cd, Cu, and Ni sensitivity was revealed. In summary, both *HERK* genes are likely to be important to support root growth upon higher Cd, Cu, and Ni concentrations while the Zn response is *HERK1* specific and the Pb response is *HERK2* specific.

As with wild type the hypocotyl elongation in the mutants differs from root growth responses. Already on control medium *herk1* elongates significantly less than wild type and the two *herk2* alleles (**Figure 3C** and **Table 1**). Therefore, normalization to control conditions was necessary to reveal that hypocotyl elongation of *herk1* was less disturbed by Cd, Ni, Zn, and Pb than wild type (**Figure 3D** and **Table 1**). Identical responses were found in the two *herk2* mutants (**Figures 3C,D** and **Table 1**). In conclusion, both *HERK* genes appear to mediate the inhibition of hypocotyl elongation upon Cd, Ni, Zn, and Pb while they support root growth on Cd, Cu, and Ni.

### THESEUS1 Is Mediating Ni Specific Inhibition of Hypocotyl Elongation in the Dark without Cell Wall Damage Responses

*THE1* signaling negatively affects cell expansion upon inhibition of cellulose synthesis (Hématy et al., 2007; Merz et al., 2017). Therefore *THE1* is the strongest candidate to uncover metal ion induced cell wall damage responses. Well-characterized loss-of-function and gain-of-function alleles were available to compare their responses to metal ions.

**FIGURE 4:**
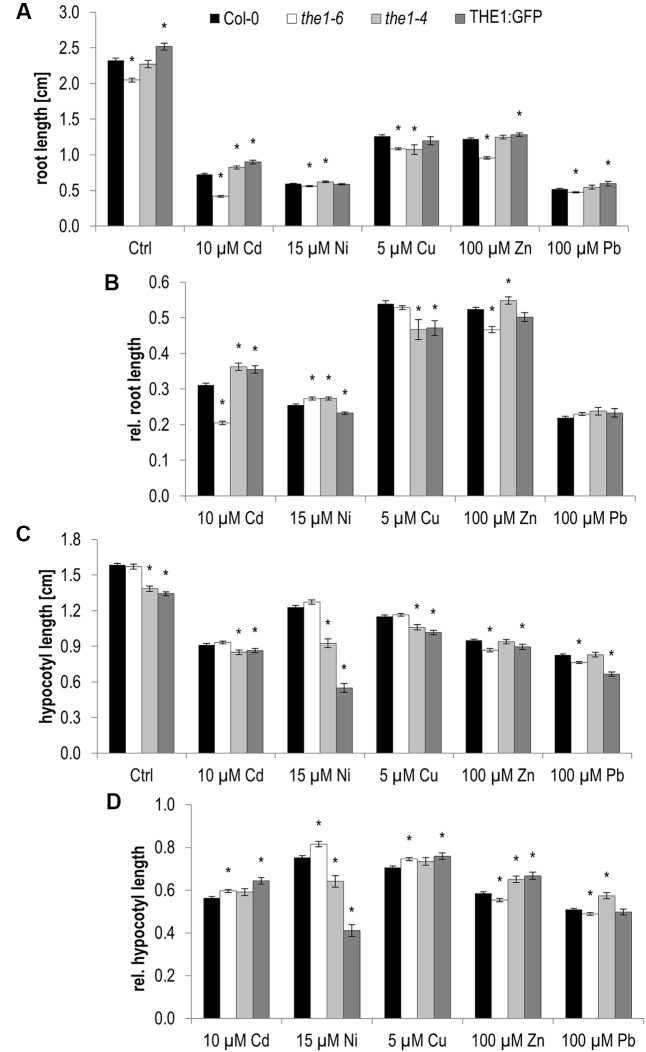
Root growth and etiolated hypocotyl elongation of different *the1* alleles on medium supplemented with high metal ion concentrations at 5 days after germination. **(A)** Absolute root lengths, **(B)** relative root lengths, **(C)** absolute hypocotyl lengths, **(D)** relative hypocotyl lengths. Shown are mean and SE of at least three biological replicates with 20 seedlings each. Stars indicate significant differences according to Student’s *t*-tests with ^∗^*p* < 0.05 to wild type **(A,C)**, wild type and control medium **(B,D)**.

Already on control medium root growth of *THE1*-related knock-out and overexpression lines behaved inversely (**Figure 4A** and **Table 1**). While the loss-of-function mutant *the1-6* developed shorter roots, roots of the THE1:GFP overexpressor grew faster than wild type. Similar contrasting root growth responses were significant on Cd and Ni. On Cu only THE1:GFP and the hypermorphic allele *the1-4* developed shorter roots as did *the1-6* on Zn (**Figure 4B** and **Table 1**).

We previously showed that the hypermorphic *the1-4* expresses a transcript encoding a predicted membrane-associated truncated protein lacking the kinase domain. Differences between THE1:GFP and the hypermorphic *the1-4* allele, as seen on control, Ni and Zn, possibly relate to the missing cytoplasmic domain in *the1-4* which might be important for feedback regulation (Merz et al., 2017).

**FIGURE 5:**
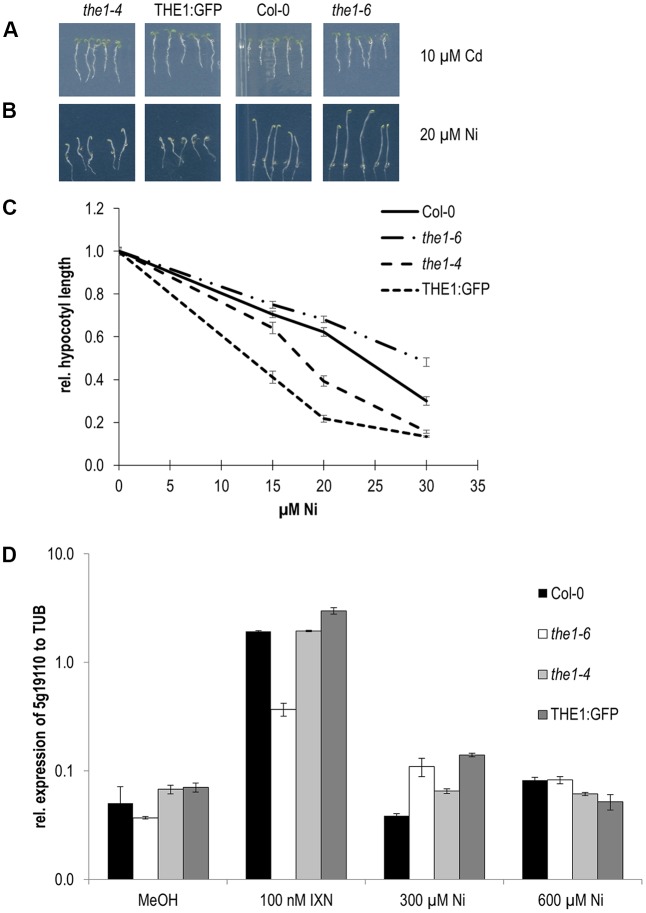
Responses of THE1 alleles to Cd and Ni. **(A)** Root growth on Cd, **(B)** etiolated hypocotyl elongation on Ni, **(C)** dose responses of Ni on etiolated hypocotyls, **(D)** expression of a downstream gene of the THE1 mediated cell wall damaging signaling pathway upon treatment with the cellulose biosynthesis inhibitor isoxaben (IXN), mock (MeOH) and different concentrations of Ni.

Hypocotyl elongation in the dark behaved different to roots for *the1-6* on control Cd, and Pb, for THE1:GFP on control, Cu and Zn and for *the1-4* on all conditions (**Figures 4C,D** and **Table 1**). While in roots the strongest effect was shown by *the1-6* on Cd, for hypocotyls it was on Ni by *the1-4* and THE1:GFP (**Figure 5A**). The opposing effects of the loss- and gain-of function alleles on hypocotyl elongation were even more distinct on 20 and 30 μM Ni (**Figures 5B,C**). These results indicate that THE1 mediates Ni specific inhibition of hypocotyl elongation in the dark and promotes Cd specific growth tolerance in roots.

As mentioned above *THE1* is known to inhibit cell elongation in hypocotyls upon cell wall damage. Therefore, we examined if Ni induces cell wall damage specific responses such as the expression of the downstream gene in the *THE1* signaling pathway, *EDGP/At5g19110*. The expression of *At5g19110* was strongly induced in the overexpressor and the hypermorphic alleles and did not respond in the loss-of-function allele, *the1-6* in chemically induced cell wall damage by treatment with the cellulose biosynthesis inhibitor isoxaben. Upon Ni treatment a very low expression in the different *THE1* alleles was quantified with no significant alterations to wild type (**Figure 5D**). These data suggest that Ni perception by THE1 is independent of cell wall damage specific gene expression responses.

### FERONIA Mediates Growth Inhibition of Hypocotyl on Cd, Cu, Pb, and Zn While it Promotes Growth on Ni

*FER* is the best studied member of the *CrRLK1L* gene family. *FER* has been identified due to its crucial function during pollen-tube ovule interaction (Escobar-Restrepo et al., 2007) but is also important for bacterial pathogen interaction (Keinath et al., 2010; Stegmann et al., 2017), mechanosensing (Shih et al., 2014) and in the antagonistic cross-talk between ABA and auxin in vegetative tissues (Yu et al., 2012). Mutants of *FER* exhibit diverse pleotropic phenotypes. They are stunted, develop smaller leaves (Guo et al., 2009b) and shorter etiolated hypocotyls which are hypersensitive to ethylene and insensitive to brassinosteroids (Deslauriers and Larsen, 2010). Moreover *fer* mutants develop shorter root hairs (Duan et al., 2010; Du et al., 2016) and larger seeds (Yu et al., 2014) and exhibit a reduced gravitropic response which correlates with less apoplastic alkalization at the lower side of gravistimulated roots (Barbez et al., 2017).

**FIGURE 6:**
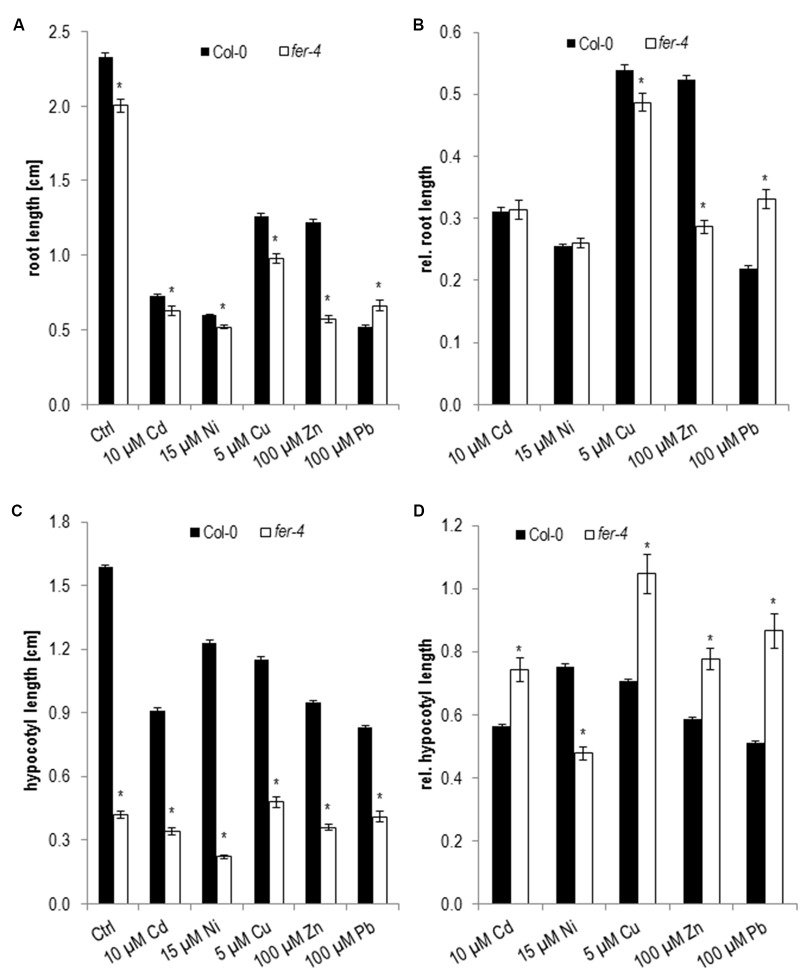
Root growth and hypocotyl elongation of *fer-4* on medium supplemented with elevated metal ion concentrations at 5 dag. **(A)** Absolute root length, **(B)** corresponding relative root lengths, **(C)** absolute hypocotyl lengths, **(D)** corresponding relative hypocotyl lengths. Shown are mean and SE of at least three biological replicates with 20 seedlings each. Stars indicate significant differences according to Student’s *t*-tests with ^∗^*p* < 0.05 to wild type **(A,C)**, wild type and control medium **(B,D)**.

In our growth analyses root lengths of 5 days old *fer-4* seedlings were significantly shorter on control medium and in most metal ion treatments except on Pb (**Figure 6A** and **Table 1**). However when root growth was normalized to control conditions *fer-4* was hypersensitive to Zn and Cu while it was more tolerant to Pb (**Figure 6B** and **Table 1**). In concordance with published data etiolated *fer-4* hypocotyl reached only 25% of the size of wild type (**Figure 6C** and **Table 1**). Thus the different effects of the metal ions were revealed after normalization to control conditions (**Figure 6D** and **Table 1**) and showed that *fer-4* is hypersensitive to Ni and has a higher tolerance to all other tested metal ions.

### Medium Alkalinization of Etiolated Seedlings Is Constitutive in *fer-4* Hypocotyls and Ni Specific in the THE1 Overexpressor

FER binds and is activated by secreted peptides of the RALF family (Haruta et al., 2014) triggering alkalinization of the apoplast. This alkalinization is thought to counteract the acidification crucial for cell expansion. Light grown *fer-4* seedlings acidified a bath medium faster than wild type which is indicative for a higher plasma membrane H^+^-ATPase activity (Haruta et al., 2014).

**FIGURE 7:**
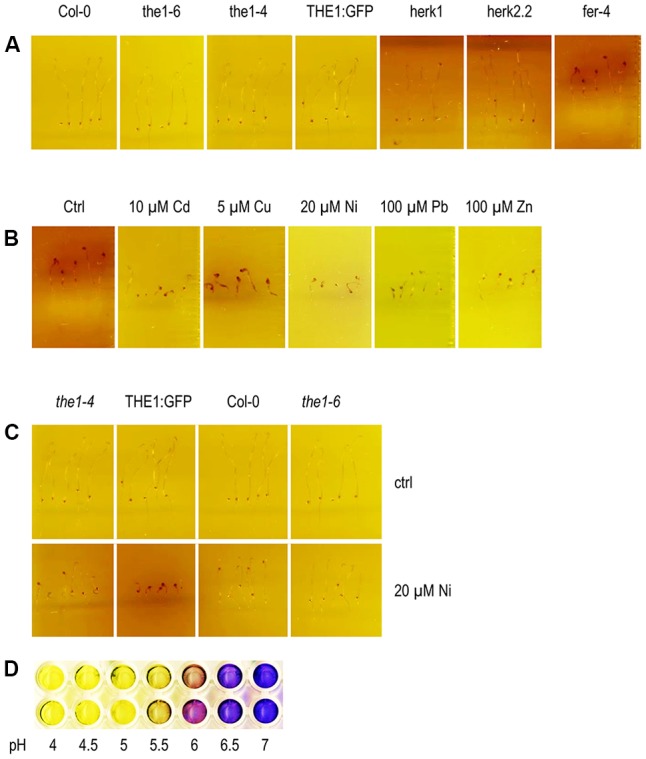
Alkalinization of the medium around etiolated hypocotyls. **(A)** On control medium, **(B)** of *fer-4* on metal ions and **(C)** of loss- and gain-of-function alleles of *THE1* on elevated Ni concentrations. **(D)** BCP is yellow below pH < 5.2 and purple above pH > 6.0. The contrast and brightness of the pictures were increased by 50% and 20%, respectively, except for *fer-4* on Pb where only the contrast was raised to +50%.

Since growth of etiolated hypocotyl depends on rapid cell expansion we tested the acidification capacity of the *Cr*RLK1L mutants on control medium and upon metal ion treatments (**Figures 7A–C**). All genotypes acidified the medium supplemented with the pH indicator bromocresolpurple (BCP) (**Figure 7D**). Only *fer-4* exhibited a remarkable alkalinization around the hypocotyl while the root was still acidifying the medium (**Figure 7B**). This alkalinization might be one of the reasons why etiolated hypocotyls of *fer-4* are short and indicate that the plasma membrane H^+^-ATPase activity is differently regulated from roots. The phenotype further suggests that *FER* is necessary for the acidification of etiolated hypocotyls. It is conceivable that in the absence of *FER* *RALF* expression is triggered and another receptor in the hypocotyl mediates the alkalinization. The alkalinization surrounding the *fer-4* etiolated hypocotyl was still visible on medium supplemented with metal ions. Hypoctoyl mediated alkalinization of the medium is strongest on control medium, followed by Cu, Ni, and only slightly on Cd and Zn. Since, the medium supplemented with Pb has a lower pH itself the alkalinization takes longer. The only other genotype with reduced acidification capacity was the overexpressor THE1:GFP on Ni (**Figure 7C** and Supplementary Figure 1). Similar to *fer-4*, THE1:GFP developed shorter hypocotyls on Ni. It might be possible that overexpression of THE1:GFP increases the sensitivity to Ni induced hypocotyl specific RALFs and that THE1 might be involved in their signaling pathway.

## Conclusion

**FIGURE 8:**
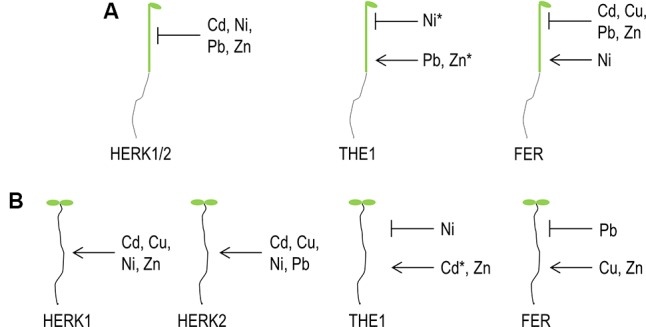
Graphical summary of *Cr*RLK1L functions on elevated metal ion concentrations. **(A)** Etiolated hypocotyl elongation, **(B)** root growth. Arrows indicate that the particular *Cr*RLK1L protein is required to maintain growth while cross bars illustrate an inhibitory function upon specific metal ions. ^∗^ Indicate opposite effects of *the1-6* loss-of-function versus gain-of-function alleles (*the1-4*, THE1:GFP) on elevated concentrations of the marked metal ion.

Our survey revealed a molecular link between metal ion stress, growth and the cell wall integrity sensors of the *Cr*RLK1L family. By analyzing growth responses in roots and hypocotyls a complex network of patterns of gene specific, overlapping and antagonistic responses which are summarized in **Table 1** and **Figure 8** was uncovered. Few general patterns were extractable of the data: in all *HERK1*, *HERK2* and *FER* mutants root growth is affected inversely to hypocotyl elongation. This tendency is also true for the different THE1 alleles but for example on Zn in the opposite direction. The hypocotyl of the gain-of-function and hypermophic alleles of *THE1* behaved similar to *HERK1*, *HERK2*, and *FER* while the loss-of-function allele exhibited hypersensitivity toward Zn (**Table 1**). The difference of the metal ion mediated growth responses might arise as a result of the presence of different ligands in roots and fast expanding etiolated hypocotyls. One of the candidate ligands might be members of the large *RALF* gene family. RALF1 has been shown to bind and activate FER leading to the phosphorylation and inhibition of the plasma membrane H^+^-ATPase AHA2, reduction of the apoplast/cell wall acidification and the inhibition of cell expansion genes (Haruta et al., 2014). It is possible that other RALFs with different expression and pro-peptide processing patterns are triggering similar reactions in conjunction with other *Cr*RLK1Ls (Wolf and Höfte, 2014). Other possible ligands related to the putative carbohydrate binding feature of the extracellular malectin-like domain might be metal ions loaded pectin/homogalacturonans or their degradation products, oligogalacturonans. Boisson-Dernier et al. (2011) proposed for the pollen specific *Cr*RLK1L member, ANXUR1 and 2 (ANX1 and ANX2) a possible interaction between homogalacturonans. This hypothesis was based of the premature bursting of pollen tubes in the double mutants of *anx1/anx2* and the pollen specific expression of pectin modifying enzymes which correlated with a specific de-/methylesterification at the pollen tube tip and thus mechanical property. A related hypothesis was presented by Wolf and Höfte (2014) proposing a feedback loop between RALF and pectin modifying enzymes. RALF induced alkalinization of the cell wall would activate PMEs which upon removal of the methyl groups lead to cell wall acidification and cell expansion. Based on this hypothesis, metal ions might be complexed by demethylesterified pectins and stiffens the cell wall prematurely before cell expansion is completed. The puzzle why different metal ions trigger specific *Cr*RLK1Ls and why and how their signaling outcome induces opposite effects on growth remains to be solved in the future.

## Author Contributions

JR and M-TH designed the study and JR, MP, and GM performed the experiments and JR, M-TH, and LG analyzed the data. M-TH and JR wrote the manuscript.

## Conflict of Interest Statement

The authors declare that the research was conducted in the absence of any commercial or financial relationships that could be construed as a potential conflict of interest.
