# Supplementary material for: Role of CrRLK1L Cell Wall Sensors HERCULES1 and 2, THESEUS1, and FERONIA in Growth Adaptation Triggered by Heavy Metals and Trace Elements
Source: Front Plant Sci. 2017 Sep 7;8:1554. doi: 10.3389/fpls.2017.01554 (PMC5594065; doi:10.3389/fpls.2017.01554)
Supplement: Supplementary file 1 [file Presentation_1.pdf]

Supplementary Tables and Figures

Role of CrRLK1L cell wall sensors HERCULES1 and 2, THESEUS1, and FERONIA in growth adaptation triggered by heavy metals and trace elements

Supplementary Table 1 Medium compositions

**Hoaglands medium**	**mg/L**	**1x**	**1/10**
KNO3	606.6	6 mM	0.6 mM
Ca(NO3)2	656.4	4 mM	0.4 mM
MgSO4 anhydrous	240.8	2 mM	0.2 mM
NH4H2PO4	115.03	1 mM	0.1 mM
H3BO3	2.86	46 µM	4.6 µM
Na2 EDTA·2H2O	3.35	12 µM	1.2 µM
MoO_3	0.016	11 µM	1.1 µM
FeSO4·7H2O	2.5	8 µM	0.8 µM
MnCl2·4H2O	1.81	9 µM	0.9 µM
ZnSO4.7H2O	0.22	0.77 µM	0.077 µM
CuSO4.5H2O	0.08	0.32 µM	0.032 µM

**MS medium**	**mg/L**	**1x**
CaCl2	332.0	3 mM
KH2PO4	170	1.3 mM
KNO3	1900	18.8 mM
MgSO4	180.5	1.5 mM
NH4NO3	1650	20.6 mM
CoCl2.6H2O	0.025	0.1 µM
CuSO4.5H2O	0.025	0.1 µM
FeNaEDTA	36.7	100 µM
H3BO3	6.2	100.3 µM
KI	0.83	5.0 µM
MnSO4.H2O	16.9	100 µM
Na2MoO4.2H2O	0.25	1.0 µM
ZnSO4.7H2O	8.6	29.9 µM

Supplementary Table 2 Primers for RT-qPCR

Gene name	AGI number	forward 5'-3'	reverse 5'-3'
**AP2M** clathrin adaptor complex subunit	At5g46630	GTTTGGGAGAAGAGCGGTTA	CTGATGTCACTGAACCTGAACTG
**FER**	At3g51550	AGTTTGCTGAAACCGCGATG	TGGAGCTGCAACGCAAATTC
**THE1**	At5g54380	TTCGGTGGTCTTTGTCAACG	AACGCCAAAGCTTGATCAGG
**HERK1**	At3g46290	TTCAGCCATTGGTTCGTTGC	TCGAAAACGGCATCCAAGTC
**EDGP** dermal glycoprotein- like	At5g19110	CTACAATGCTCTTGCTCAGTC	ACCGTCGATAAACGCCAAAC
**TUB9**	At4g20890	GTACCTTGAAGCTTGCTAATCCTA	GTTCTGGACGTTCATCATCTGTTC

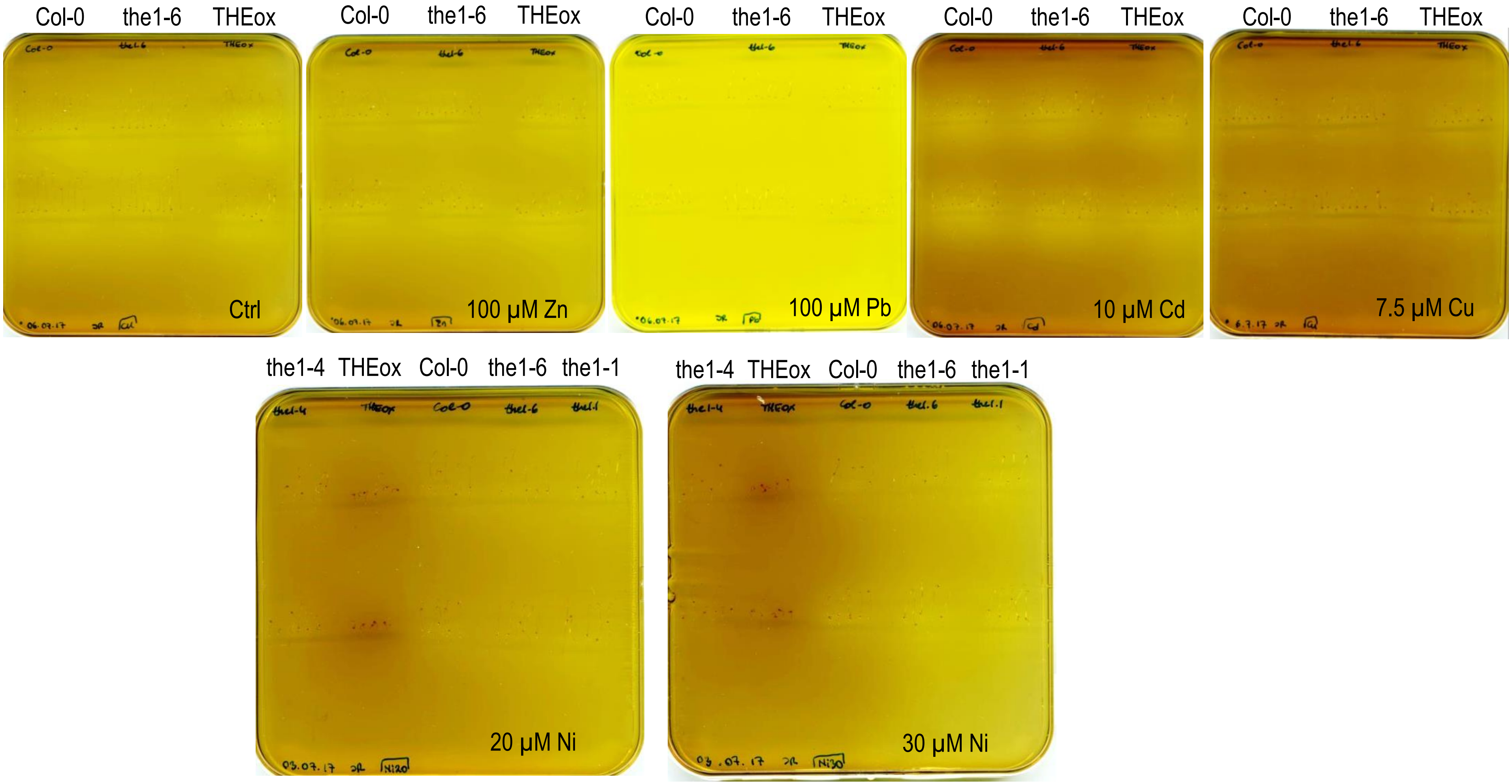

Figure S1 pH indicator **e**xperiments of THE1 alleles on control and metalloid containing media.
